# Supplementary material for: Altered dendritic spine function and integration in a mouse model of fragile X syndrome
Source: Nat Commun. 2019 Oct 23;10:4813. doi: 10.1038/s41467-019-11891-6 (PMC6811549; doi:10.1038/s41467-019-11891-6)
Supplement: Supplementary file 1 — Supplemental Information [file 41467_2019_11891_MOESM1_ESM.pdf]

Altered dendritic spine function and integration in a mouse model of Fragile X Syndrome

Booker SA et al.,

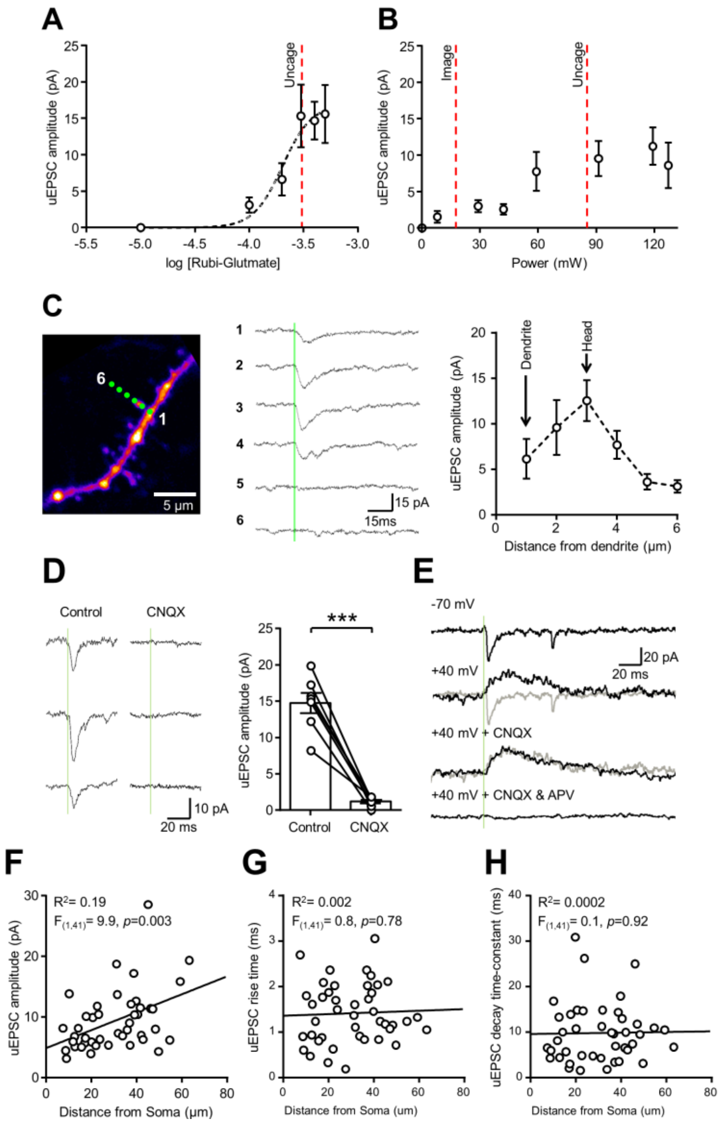

Supplementary Figure 1: Characterisation of glutamate uncaging and uEPSC properties of uEPSCs produced at dendritic spines on L4 SCs. **A** Dose-response curve for Rubi-glutamate following 1 ms activation (780 nm, 80 mW, 1 µm distal from spine) in WT mice. Concentration used for experiments in Fig. 1 indicated (red line). **B** Power-response relationship of 300 µM Rubi-glutamate. Laser power used for imaging and uncaging are indicated (red lines). **C** Spatial photolysis characterised as a function of distance from dendrite shaft, in 1 µm. Average spatial profile of 10 spines from WT cells (right). **D** uEPSCs recorded at -70 mV were fully blocked by the AMPAR antagonist CNQX (10 µM; d.f.: 6; t = 9.42; p < 0.0001; paired T-test). **E** Electrical and pharmacological characterization of uEPSCs recorded at -70 mV (top) and +40 mV (lower panels). uEPSCs at +40 mV were minimally sensitive to CNQX (10 µM), but blocked by APV (50 µM); confirming NMDAR uEPSCs. **F** comparison of uEPSC amplitude as a function of distance from soma in WT L4 SCs. Linear regression values are shown confirming increased

synaptic amplitude in distal synapses. G No change in uEPSC rise-time,as measured as the 20-80% of the uEPSC peak. **H** No change in uEPSC decay time-constant as a function of distance from soma. Statistics shown: *** – $p < 0.001$, T-test (D) and sum of least squares F-test (F-H). Data shown as mean ± SEM.

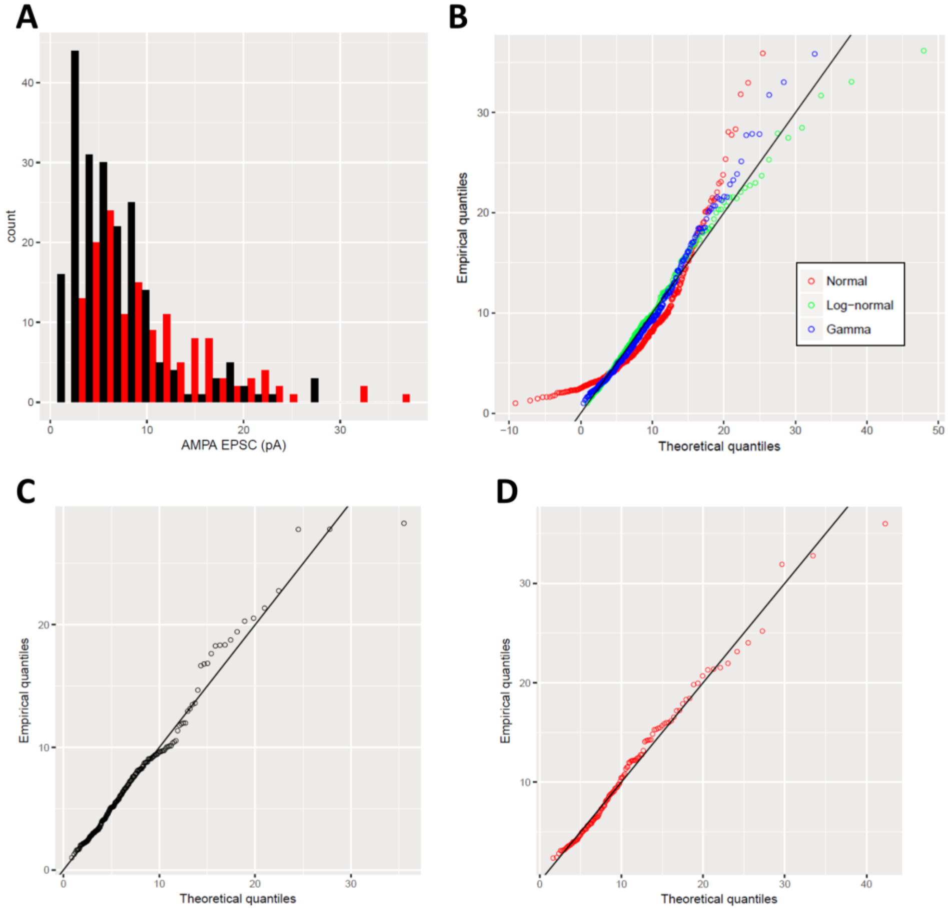

E

AMPA uEPSC	Normal	Log-Normal	Gamma
Goodness-of-fit statistics			
Kolmogorov-Smirnov statistic	0.131	0.028	0.055
Cramer-von Mises statistic	2.427	0.042	0.334
Anderson-Darling statistic	14.272	0.323	2.140
Goodness-of-fit criteria			
Akaike's Information Criterion	2227.3	2028.9	2050.9
Bayesian Information Criterion	2235.0	2036.6	2058.6

F

	Model
fit_0	1 + (1|animal) + (1|cell)
fit_genotype	genotype + (1|animal) + (1|cell)

AMPA uEPSC	DF	AIC	BIC	logLik	deviance	Chisq	Chi DF	Pr (>Chisq)
fit_0	4	640.6	656.0	-316.3	632.6			
fit_genotype	5	634.3	653.6	-312.2	624.3	8.2547	1	**0.0041**

Supplementary Figure 2: Linear mixed effects models (LMMs) of AMPA uEPSC amplitudes. **A** Binned frequency histogram data of uEPSCs of dendritic spines from WT (black) and *Fmr1*$^{-/y}$ (red). **B** Quantile data of uEPSCs compared to expected quantiles of normal (red), log-normal (green) and gamma (blue) distributions. **C, D** Quantile comparison of a log-normal distribution split by genotype in WT (black) and *Fmr1*$^{-/y}$ (red) spines. **E** Summary of goodness-of-fit for tested models. **F** Summary of LMMs (top) and comparison of genotype as the fixed effect (bottom).

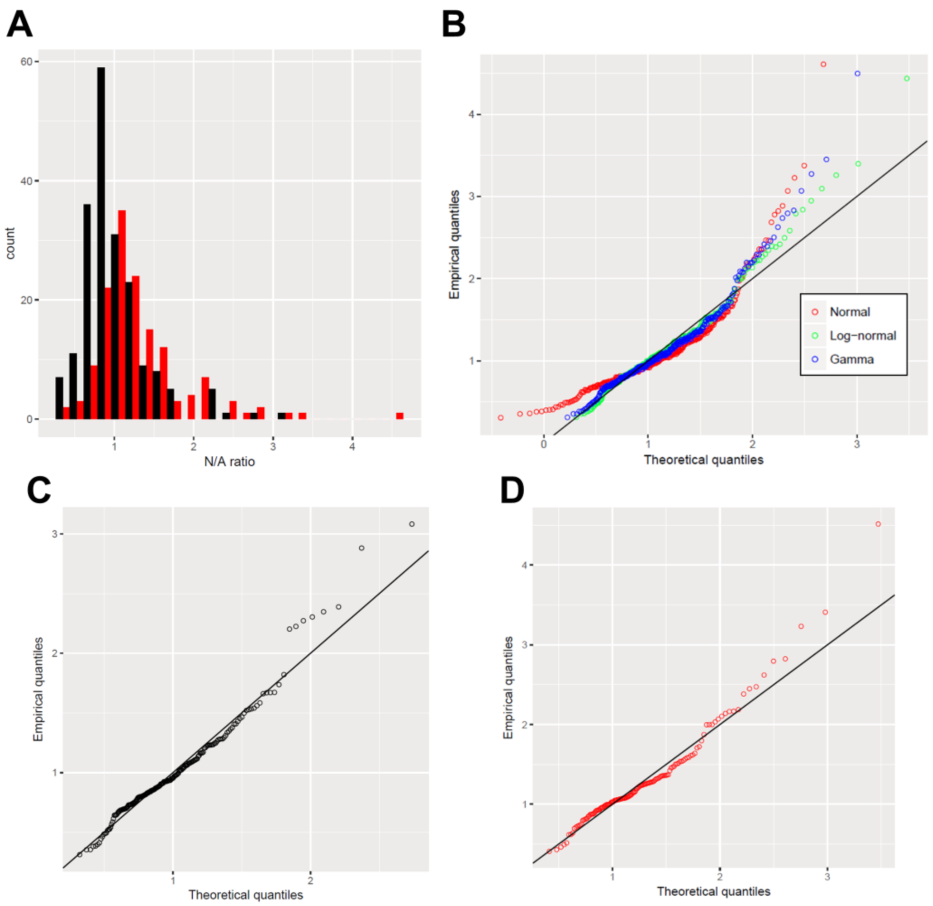

E

NMDA/AMPA ratio	Normal	Log-Normal	Gamma
Goodness-of-fit statistics			
Kolmogorov-Smirnov statistic	0.141	0.057	0.084
Cramer-von Mises statistic	2.248	0.299	0.651
Anderson-Darling statistic	13.012	1.928	3.866
Goodness-of-fit criteria			
Akaike's Information Criterion	526.8	384.6	407.4
Bayesian Information Criterion	534.4	392.2	415.1

F

	Model
fit_0	1 + (1|animal) + (1|cell)
fit_genotype	genotype + (1|animal) + (1|cell)

N/A ratio	DF	AIC	BIC	logLik	deviance	Chisq	Chi DF	Pr (>Chisq)
fit_0	4	306.8	322.1	-149.4	298.8			
fit_genotype	5	302.5	321.7	-146.2	292.5	6.2704	1	**0.0123**

Supplementary Figure 3: LMMs of NMDA/AMPA ratios. **A** Binned frequency histogram data of NMDA/AMPA ratio at dendritic spines from WT (black) and *Fmr1*$^{-/y}$ (red). **B** Quantile data of NMDA/AMPA ratio compared to expected quantiles of normal (red), log-normal (green) and gamma (blue) distributions. **C, D** Quantile comparison of a log-normal distribution split by genotype in WT (black) and *Fmr1*$^{-/y}$ (red) spines. **E** Summary of goodness-of-fit for tested models. **F** Summary of LMMs (top) and comparison of genotype as the fixed effect (bottom).

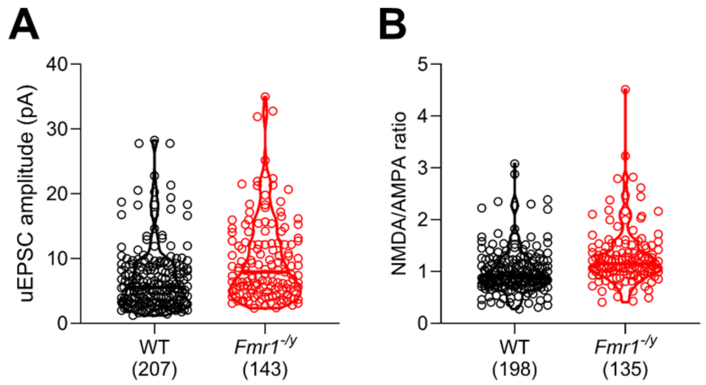

Supplementary Figure 4: Summary of all cell replicates as shown in Figure 1. **A** Violin plots of uEPSC amplitude replicates shown as a barchart in Figure 1B, plotted for WT (black) and *Fmr1*$^{-/y}$ (red) mice, overlain by data from all recorded dendritic spines. The median is indicated by a horizontal line and number of spines indicated in parenthesis. **B**, NMDA/AMPA ratios shown in Figure 1H, plotted according to the same scheme as A.

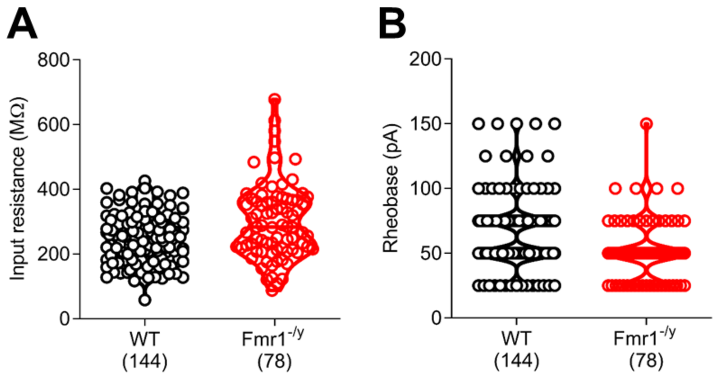

Supplementary Figure 5: Summary of individual cell properties, as reported in Figure 6. **A**, Violin plot and individual cell data (open circles) as shown in Figure 6C (inset), as reported for WT (black) and *Fmr1*$^{-/y}$ (red) neurons. Median is shown as a horizontal line and the number of recorded cells shown in parenthesis. **B** Individual cell data, plotted according to the same scheme as **A**, but for rheobase as presented in Figure 6D (inset).

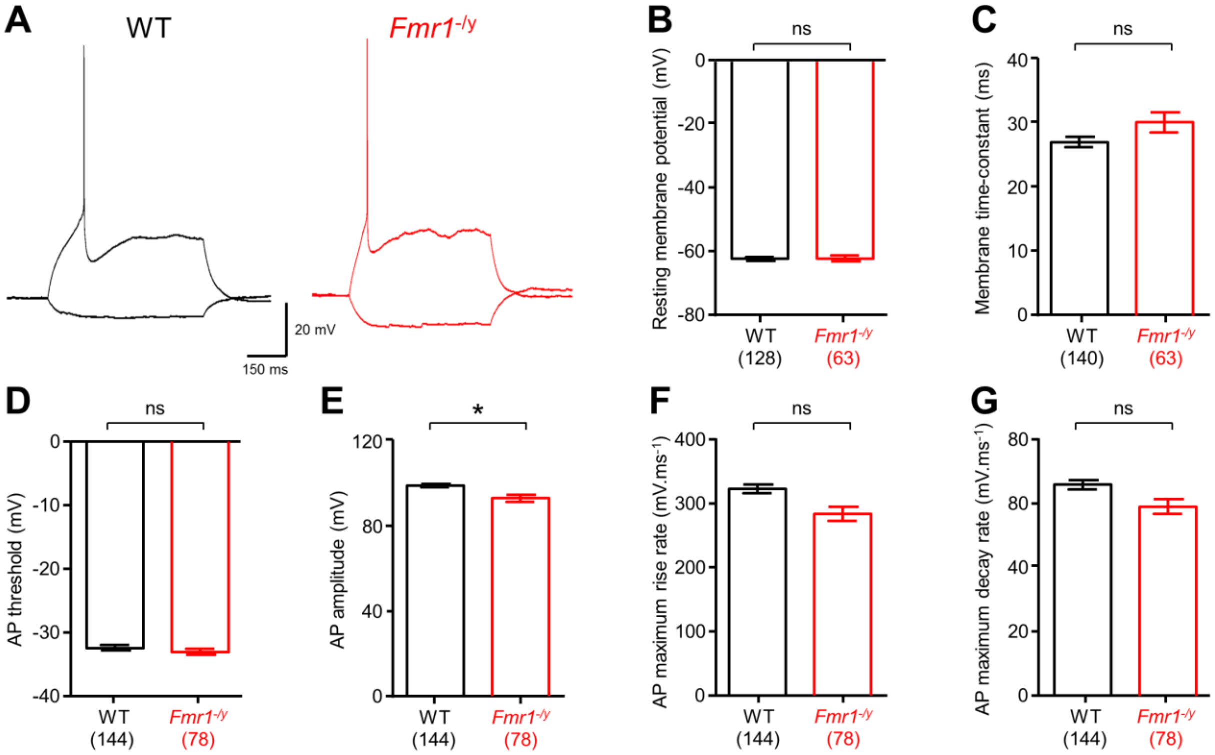

Supplementary Figure 6: Characterisation of intrinsic physiological properties of L4 stellate cell: **A** Representative hyperpolarising (-10 pA) and depolarising (rheobase) current steps (500 ms duration), in WT (black) and *Fmr1*$^{-/y}$ (red) L4 SCs. **B-G** Quantification of passive and active intrinsic physiological properties, either from resting membrane potential (B, C) or from the first AP at rheobase (D-G). Number of cells recorded indicated in parenthesis**.** Statistics shown: ns – $p > 0.05$ * - $p < 0.05$, all from LMM. All data is shown as mean ± SEM and source data for all plots are provided as a Source Data file.

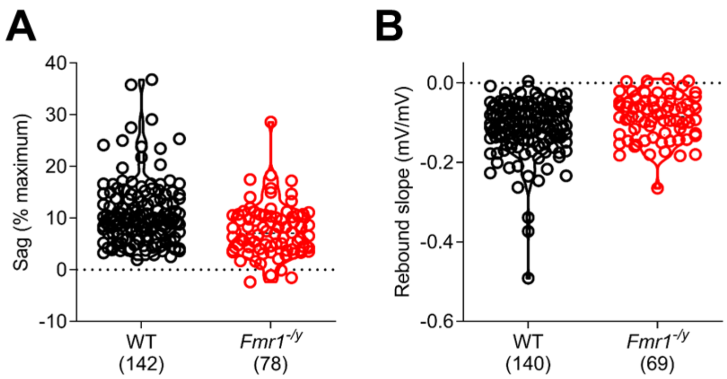

Supplementary Figure 7: Individual cell data for barcharts shown in Figure 7. **A**, Violin plot and individual cell data (open circles) for voltage sag as shown in Figure 7B reported for WT (black) and *Fmr1*$^{-/y}$ (red) neurons. Median is shown as a horizontal line and the number of recorded cells shown in parenthesis. **B** Individual cell data, plotted according to the same scheme as **A**, but for rebound slope presented in Figure 7D.

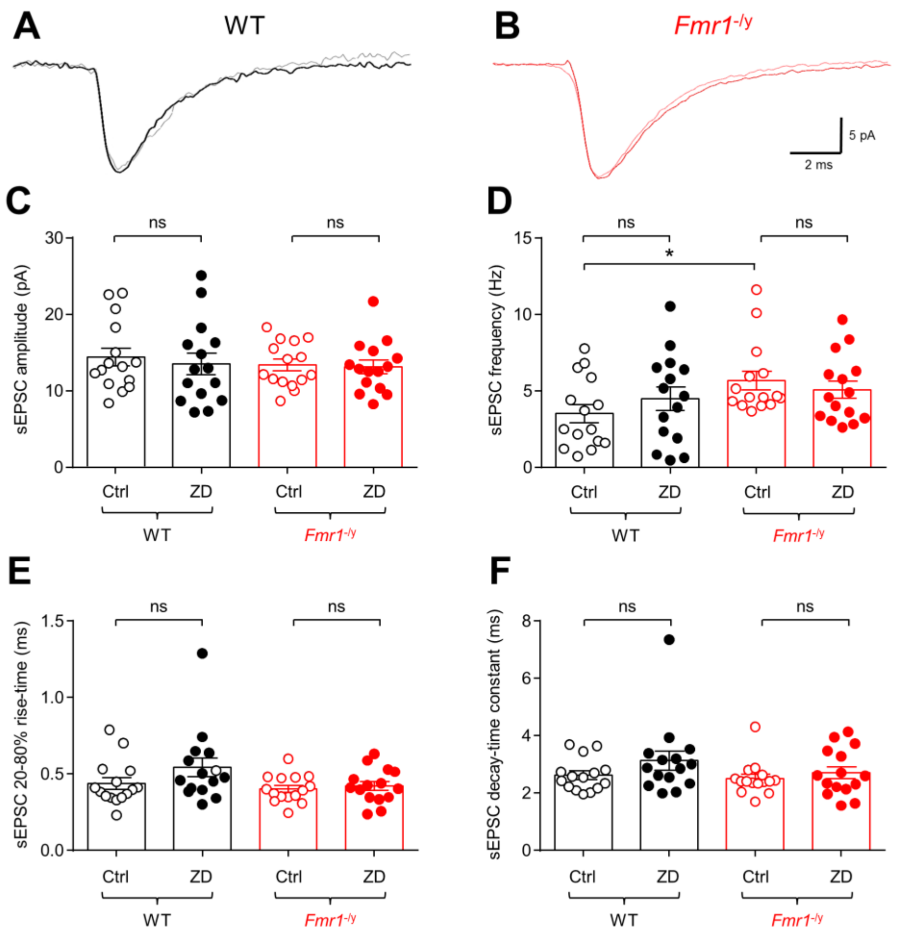

Supplementary Figure 8: Characterisation of the effect of 20 µM ZD-7,288 on spontaneous EPSCs (sEPSC). Representative sEPSCs recorded from WT (**A,** black) and *Fmr1*$^{-/y}$ (**B,** red) L4 SCs, before and after (open circles) bath application of ZD-7,288 (ZD). No difference in sEPSC amplitude (**C**), frequency (**D**), 20-90% rise time (**E**) or decay time-constant (**F**) were observed between genotypes. Individual data from cells overlain in the same colour scheme as **A** and **B.** Statistics shown: ns – p >0.05, * - p < 0.05, from LMM All data is shown as mean ± SEM and source data for all plots are provided as a Source Data file.

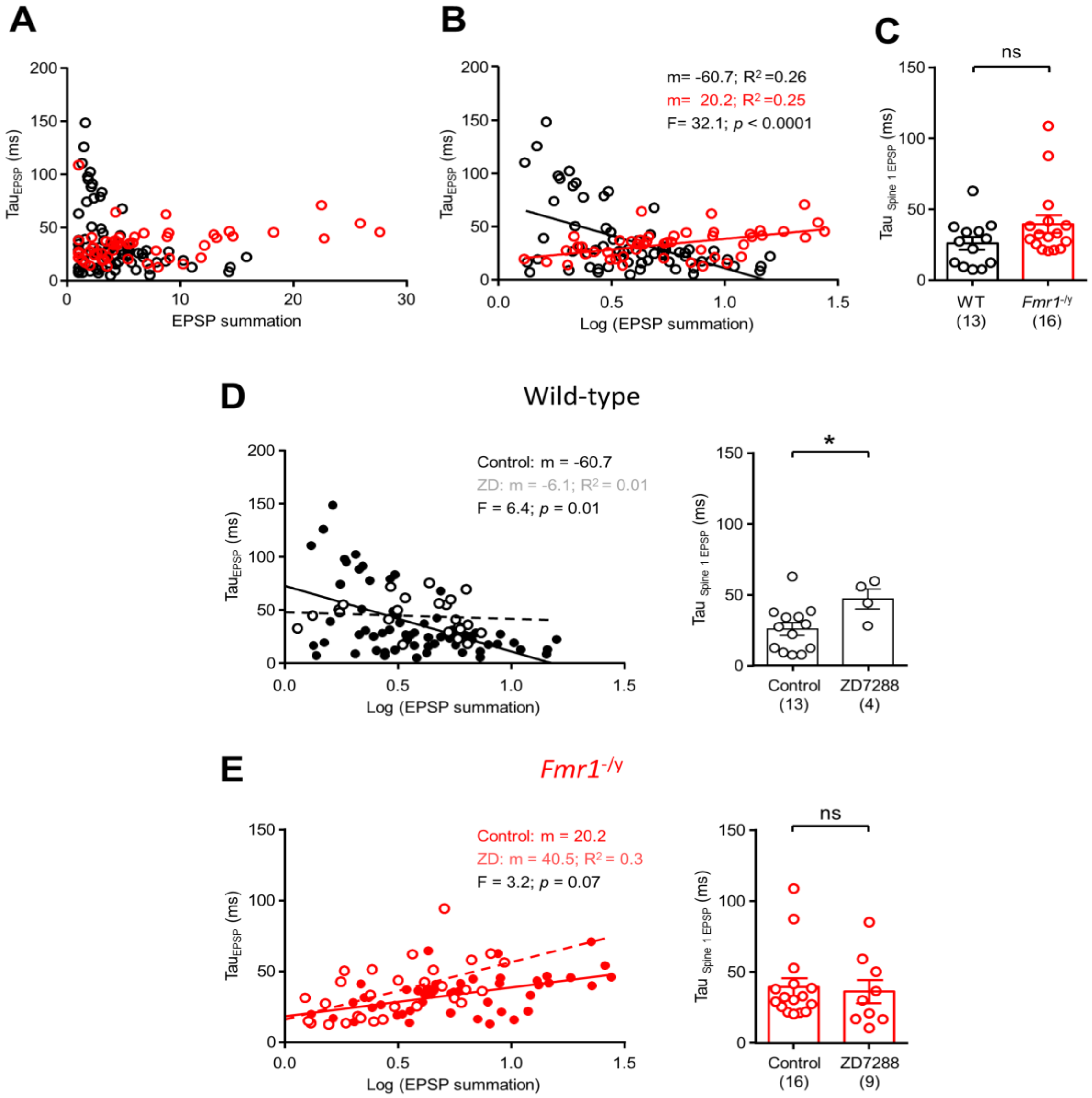

Supplementary Figure 9: Dendritic integration in WT, but not *Fmr1*-/y, mice depends on I_h. **A** uEPSP summation (normalized to 1st EPSP) and EPSP decay time-constant (Tau_{EPSP}) at dendritic spines in WT (black) and *Fmr1*-/y (red) mice. **B** The same data as **A** plotted as Log (uEPSP summation) with linear fit (lines). **C** Average of first uEPSP Tau_{EPSP}, not accounting for the observed differences in summation. **D** Block of I_h with ZD-7,288 (ZD) attenuates Tau_{EPSP} in WT mice (dashed lines, open circles).). **E** ZD application had no effect Tau_{EPSP} vs. Log (EPSP summation) in *Fmr1*-/y mice (d.f.: 1, 73; F = 3.2, p= 0.07; F-test) and no effect on Tau_{EPSP} (d.f.: 23; t = 0.31; p = 0.76; T-test). Statistics shown: ns – p> 0.05, * - P<0.05, from sum-of-least-squares F-test (B, D, E) and t-tests (C, D, E). All data is shown as mean ± SEM and source data for all plots are provided as a Source Data file.

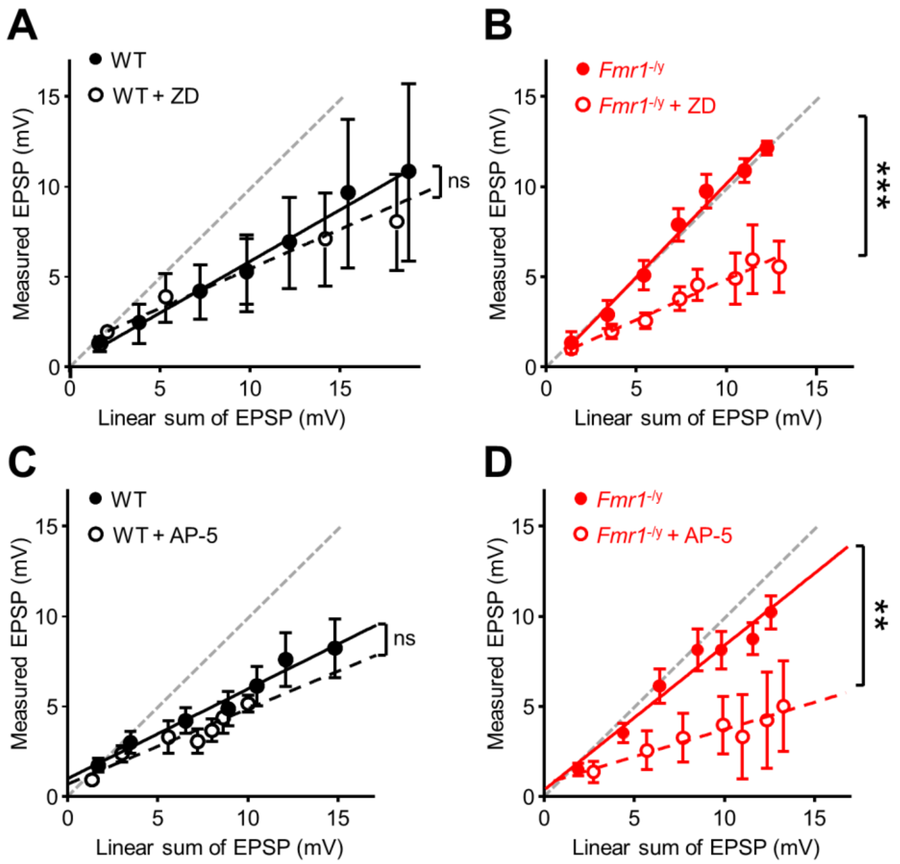

Supplementary Figure 10: HCN channels and NMDARs contribute to excessive dendritic summation in *Fmr1*$^{-/y}$ L4 SCs. **A** Observed EPSP summation plotted against the expected linear sum for WT L4 SCs (n=3 cells), under control conditions (filled circles) and following bath application of 20 µM ZD-7,288 (open circles), showing no change in summation ($d.f._{2,\ 35}$, F = 0.28, *p* = 0.75 Sum-of-least-squares F-test), **B** ZD application to summating EPSCs in *Fmr1*$^{-/y}$ L4 SCs (n=4 cells) strong reduced summation ($d.f._{2,\ 52}$, F = 54.3, *p* <0.0001, Sum-of-least-squares F-test). **C** WT L4 SCs (n=3), under control conditions (black circles) and following bath application of 50 µM AP-5 (open circles). There was no difference in the slope of linear regression between treatment ($d.f._{1,\ 98}$, F = 0.08, *p* = 0.77, Sum-of-least-squares F-test), indicating minimal effect of NMDARs on WT sublinear summation. **D** AP-5 application to summating EPSPs in *Fmr1*$^{-/y}$ L4 SCs resulted in a substantial reduction in the observed summation ($d.f._{1,\ 109}$, F = 7.85, *p* = 0.006, Sum-of-least-squares F-test), indicating that NMDARs are involved in the enhanced summation observed. Statistics shown: ns – *p* >0.05, ** - *p*<0.01, *** *p*<0.0001, from sum-of-least-squares F-test. All data is shown as mean ± SEM and source data for all plots are provided as a Source Data file.

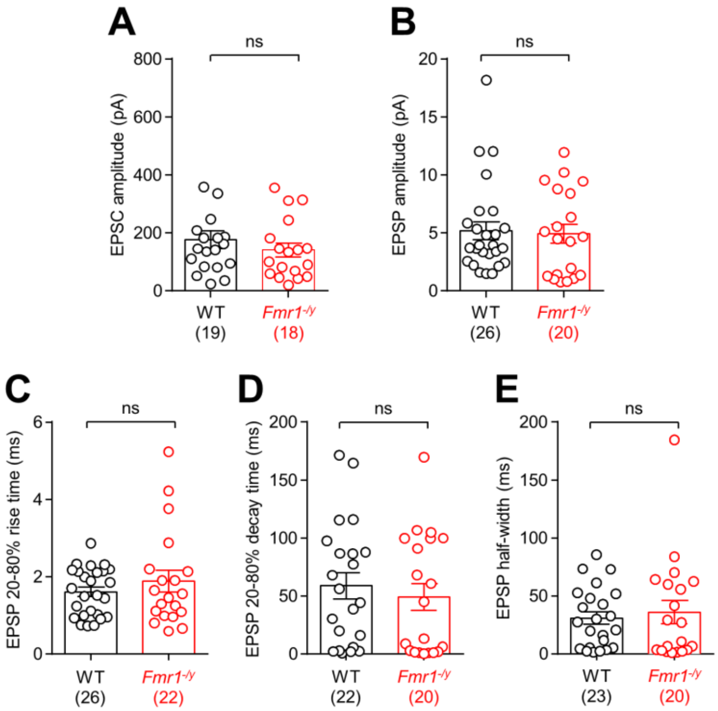

Supplementary Figure 11: Properties of TCA synaptic responses in L4 SCs. **A** quantification of evoked EPSCs following stimulation of the ventrobasal thalamus and recruitment of TCA synapses from WT (n=19) and *Fmr1*$^{-/y}$ L4 SCs showing no difference in overall synaptic strength (d.f..= 35, t = 0.9, p = 0.7). **B** There was no difference in amplitude of the resulting EPSP between genotypes (d.f..= 44, t = 0.2, p = 0.8). **C** EPSP 20-80% rise time (d.f..= 44, t = 1.0, p = 0.3), **D** 20-80% decay time (d.f..= 40, t = 0.6, p = 0.6), nor EPSP half-width **E** (d.f..= 41, t = 0.5, p = 0.7) showed any major difference between genotype. Statistics shown: ns – p >0.05, all from Mann-Whitney tests. All data is shown as mean ± SEM and source data for all plots are provided as a Source Data file.

Figure	Comparison	Mean ± SEM	*n*	Mean ± SEM	*n*	Replicate	Test	Distribution	Model	*t*/U/F	*p*
1B	WT vs. *Fmr1*$^{-/y}$	6.9 ± 0.4 pA	207	9.8 ± 0.5 pA	143	Spine	LMM	Log-normal		8.26	0.004
1C	WT vs. *Fmr1*$^{-/y}$	6.9 ± 0.6 pA	17	9.1 ± 0.9 pA	14	Mouse	Unpaired t-test	Normal		2.09	0.0460
1D	WT vs. *Fmr1*$^{-/y}$	0.47 ± 0.06 mV	16	0.73 ± 0.12 mV	10	Mouse	Unpaired t-test	Normal		2.15	0.0420
1F	WT vs. *Fmr1*$^{-/y}$	6.4 ± 1.6%,	17	17.6 ± 3.5%	13	Mouse	Unpaired t-test	Normal			0.0045
1G	WT vs. *Fmr1*$^{-/y}$	0.76 ± 0.03	200	1.05 ± 0.04	135	Spine	F-test	Linear	Slope	37.36	< 0.0001
1H	WT vs. *Fmr1*$^{-/y}$	0.97 ± 0.03	194	1.26 ± 0.05	117	Spine	LMM	Log-normal		6.27	0.012
2C	WT vs. *Fmr1*$^{-/y}$	33.6%(37/110)	110	14.8% (8/54)	54	Cell pair	Fisher's Exact	Contingency			0.0149
2D	WT vs. *Fmr1*$^{-/y}$	27.7 ± 5.0%	35	31.4 ± 11.3%	8	Cell pair	GLMM	Gamma		0.25	0.798
2E	WT vs. *Fmr1*$^{-/y}$	12.6 ± 2.0 pA	36	10.4 ± 3.8 pA	8	Cell pair	LMM	Log-normal		1.53	0.1512
3B	WT vs. *Fmr1*$^{-/y}$	0.43 ± 0.05 µm	6	0.45 ± 0.04µm	4	Mouse	Unpaired t-test	Normal		0.29	0.7781
3C	WT vs. *Fmr1*$^{-/y}$	7.8 ± 3.8	72	7.0 ± 5.4	32	Spine	F-test	Linear	Slope	0.02	0.8925
3D	WT vs. *Fmr1*$^{-/y}$	1.52 ± 0.22 µm	6	1.31 ± 0.20 µm	4	Mouse	Unpaired t-test	Normal		0.66	0.5285
3E	WT vs. *Fmr1*$^{-/y}$	2.1 ± 0.75	72	0.8 ± 1.4	32	Spine	F-test	Linear	Slope	0.84	0.3607
3F	WT vs. *Fmr1*$^{-/y}$	6.76 ± 0.65 /10 µm	10	6.12 ± 0.80 /10µm	5	Mouse	Unpaired t-test	Normal		0.60	0.5588
3H	WT vs. *Fmr1*$^{-/y}$	0.48 ± 0.05 µm	10	0.48 ± 0.04 µm	5	Mouse	Unpaired t-test	Non-normal		20.0	0.5921
3J	WT vs. *Fmr1*$^{-/y}$	1.36 ± 0.12 µm	10	1.28 ± 0.14 µm	5	Mouse	Unpaired t-test	Non-normal		20.0	0.5541
	uEPSC vs. spine head (WT)	7.8 ± 3.8 pA/µm	70			Spine	F-test	Linear		4.79	0.0423
4C	WT vs. *Fmr1*$^{-/y}$	7.2 ± 1.5%	3	20.5 ± 1.6%	7	Mouse	Unpaired t-test	Normal		4.9	0.0010
5B (left)	WT vs. *Fmr1*$^{-/y}$	13.1 ± 0.8 pA	24	12.7 ± 1.3 pA	24	Mouse	Mann-Whitney	Non-normal		245	0.2765
5B (right)	WT vs. *Fmr1*$^{-/y}$	3.9 ± 0.5 Hz	24	4.9 ± 0.6 Hz	24	Mouse	Mann-Whitney	Non-normal		240	0.2349
5C (left)	WT vs. *Fmr1*$^{-/y}$	16.9 ± 2.6 pA	8	14.4 ± 1.6 pA	11	Mouse	Mann-Whitney	Non-normal		37	0.5931
5C (right)	WT vs. *Fmr1*$^{-/y}$	1.7 ± 0.2 Hz	8	2.6 ± 0.3 Hz	11	Mouse	Mann-Whitney	Non-normal		18	0.0325
6C	WT vs. *Fmr1*$^{-/y}$		29		19	Mouse	2-way ANOVA	Normal	Interaction	7.03	<0.0001
-25		-7.8 ± 0.4 mV		-9.8 ± 0.7 mV			+ Bonferroni			1.26	0.7568
-50		-14.6 ± 0.6 mV		-18.3 ± 1.1 mV						2.19	0.1658
-75		-20.3 ± 0.9 mV		-24.9 ± 1.6 mV						2.75	0.0371
-100		-25.5 ± 1.1 mV		-31.4 ± 2.0 mV						3.50	0.0032

-125		-30.0 ± 1.2 mV		-37.8 ± 2.8 mV						4.57	<0.0001
Inset	WT vs. *Fmr1*$^{-/y}$	301.1 ± 7.7 MΩ	145	385.7 ± 17.6 MΩ	79	Cell	GLMM	Gamma		2.21	0.0234
6D	WT vs. *Fmr1*$^{-/y}$		29		19	Mouse	RM	Normal	Interaction	6.17	<0.0001
-25		0.2 ± 0.1		0.7 ± 0.2			2-way ANOVA			0.62	0.9902
-50		1.8 ± 0.3		3.8 ± 0.6			+ Bonferroni			2.48	0.0798
-75		4.8 ± 0.5		7.5 ± 0.8						3.24	0.0081
-100		8.1 ± 0.7		11.2 ± 1.0						3.77	0.0012
-125		10.7 ± 0.8		14.3 ± 1.1						4.35	0.0001
Inset	WT vs. *Fmr1*$^{-/y}$	65.2 ± 2.3 pA	145	51.3 ± 2.6 pA	78	Cell	GLMM	Gamma		-2.15	0.0353
6F	WT vs. *Fmr1*$^{-/y}$	0.84 ± 0.06 Hz	13	1.13 ± 0.07 Hz	14	Cell	LMM	Log-normal		3.25	0.0024
7B	WT vs. *Fmr1*$^{-/y}$	10.9 ± 0.5%	142	7.6 ± 0.6%	78	Cell	GLMM	Gamma		-3.59	0.0003
7C	WT vs. *Fmr1*$^{-/y}$	-0.11 ± 0.01 mV/mV	29	-0.07 ± 0.01 mV/mV	19	Cell	F-test	Linear	Slope	5.67	0.0181
7D	WT vs. *Fmr1*$^{-/y}$	-0.11 ± 0.01	140	-0.9 ± 0.01	69	Cell	LMM	Log-normal		-2.28	0.0237
7E	Interaction		30		29	Cell	LMM	Log-normal		-2.43	0.0158
	WT vs. *Fmr1*$^{-/y}$	307.3 ± 19.6 MΩ	30	401.8 ± 30.9 MΩ	30	Cell	LMM	Log-normal		1.85	0.0783
	WT: Ctrl vs. ZD	307.3 ± 19.6 MΩ	30	457.6 ± 29.1 MΩ	29	Cell	LMM	Log-normal		6.05	1.99×10^{-7}
	Fmr1$^{-/y}$: Ctrl vs. ZD	401.8 ± 30.9 MΩ	30	467.5 ± 43.8 MΩ	29	Cell	LMM	Log-Normal		1.28	0.1988
7F	WT vs. *Fmr1*$^{-/y}$	166.0 ± 8.8%	29	121.2 ± 4.2%	30	Cell	LMM	Log-normal		-4.37	6.34×10^{-5}
7G	WT: Ctrl vs. ZD		17		16	Cell	2-way ANOVA	Normal	Interaction	3.20	0.0111
25		0 ± 0		1.4 ± 1.0			+ Bonferroni				0.9937
50		0.1 ± 0.1		6.8 ± 1.5							0.0396
75		4.5 ± 1.4		17.5 ± 2.2							<0.0001"
100		11.5 ± 2.3		23.0 ± 2.3							<0.0001"
125		19.5 ± 2.5		24.1 ± 2.8							0.3059

7H	Fmr1$^{-/y}$: Ctrl vs. ZD		17		14	Cell	2-way ANOVA	Normal	Interaction	0.23	0.9509
25		0.2 ± 0.2		0.1 ± 0.1			+ Bonferroni				>0.99
50		4.7 ± 1.7		6.1 ± 1.5							0.9958
75		12.4 ± 2.4		14.1 ± 2.4							0.9866
100		21.3 ± 2.4		21.1 ± 2.8							>0.99"
125		28.8 ± 2.3		27.0 ± 3.1							0.9852
7K	WT: Ctrl vs ZD	343.7 ± 32.0 MΩ	8	455.8 ± 27.0 MΩ	9	Cell	GLMM	Gamma		2.66	0.017
	Fmr1$^{-/y}$: Ctrl vs. ZD	437.0 ± 49.2 MΩ	7	497.0 ± 69.3 MΩ	8		GLMM	Gamma		0.83	0.411
8B	WT vs. *Fmr1*$^{-/y}$						F-test	Sigmoidal		4.58	0.0012
	$V_{1/2\ max}$	-83.9 ± 1.7 mV	29	-89.2 ± 1.4 mV	34	Cell	F-test			5.04	0.0253
	I_{Max}	-24.4 ± 1.0 pA	29	24.4 ± 1.1 pA	34	Cell	F-test			0.19	0.6614
8C	WT vs. *Fmr1*$^{-/y}$						F-test	Sigmoidal		4.58	0.0012
	ZD: WT: I_{Max}	-83.9 ± 1.7 mV	29	-89.2 ± 1.4 mV	34	Cell	F-test			5.04	0.0253
	ZD: *Fmr1*$^{-/y}$: I_{Max}	-24.4 ± 1.0 pA	29	24.4 ± 1.1 pA	34	Cell	F-test			0.19	0.6614
8E	WT vs. *Fmr1*$^{-/y}$						F-test	Sigmoidal		4.58	0.0012
	$V_{1/2\ max}$	-83.9 ± 1.7 mV	29	-89.2 ± 1.4 mV	34	Cell	F-test			5.04	0.0253
	I_{Max}	-24.4 ± 1.0 pA	29	24.4 ± 1.1 pA	34	Cell	F-test			0.19	0.6614
9C	All: WT vs. *Fmr1*$^{-/y}$	8.8 ± 0.7 spines	11	6.6 ± 0.6 spines	14	Cell	Unpaired t-test	Normal		2.34	0.0286
	Active: WT vs. *Fmr1*$^{-/y}$	8.7 ± 0.7 spines	11	5.6 ± 0.7 spines	9			Normal		3.18	0.0051
9D	WT vs. *Fmr1*$^{-/y}$	1.05 ± 0.13 mV/spine	13	1.89 ± 0.25 mV/spine	16	Cell	F-test	Linear	Slope	8.98	0.0031
9E	WT vs. *Fmr1*$^{-/y}$	0.50 ± 0.09 mV/mV	13	0.79 ± 0.08 mV/mV	14	Cell	F-test	Linear	Slope	3.18	0.0439
9G	WT vs. *Fmr1*$^{-/y}$: 5Hz	0.00 ± 0.00	8	0.19 ± 0.07	10	Cell	Unpaired t-test			2.57	0.0206
	WT vs. *Fmr1*$^{-/y}$: 10Hz	0.00 ± 0.00	8	0.55 ± 0.13	10	Cell				3.80	0.0016
Supplemental Materials:											
S1D	Control vs. CNQX	14.7 ± 1.4 pA	7	1.2 ± 0.3 pA	7	Cell	Paired t-test	Normal		9.42	<0.0001
S4B	WT vs. *Fmr1*$^{-/y}$	-62.0 ± 0.6 mV	128	-61.9 ± 0.9 mV	63	Cell	LMM	Normal		-0.32	0.7681
S4C	WT vs. *Fmr1*$^{-/y}$	27.3 ± 0.8 ms	140	30.4 ± 1.6 ms	63	Cell	GLMM	Gamma		0.46	0.6407
S4D	WT vs. *Fmr1*$^{-/y}$	-32.4 ± 0.4 mV	144	-33.0 ± 0.5 mV	78	Cell	LMM	Normal		-0.75	0.4796
S4E	WT vs. *Fmr1*$^{-/y}$	99.2 ± 0.8 mV	144	93.2 ± 1.7 mV	78	Cell	LMM	Normal		-1.97	0.0481

S4F	WT vs. *Fmr1*$^{-/y}$	325.1 ± 6.7 mV.ms^{-1}	144	285.6 ± 11.1 mV.ms^{-1}	78	Cell	LMM	Normal		-1.40	0.1582
S4G	WT vs. *Fmr1*$^{-/y}$	66.4 ± 1.4 mV.ms^{-1}	144	59.5 ± 2.3 mV.ms^{-1}	78	Cell	LMM	Normal		-0.88	0.3745
S5C	WT vs. *Fmr1*$^{-/y}$	14.4 ± 1.2 pA	15	13.9 ± 0.8 pA	15	Cell	LMM	Normal		0.464	0.6019
	WT: Ctrl vs. ZD	14.4 ± 1.2 pA	15	13.5 ± 1.4 pA	15	Cell	LMM	Normal		0.561	0.5714
	Fmr1$^{-/y}$: Ctrl vs. ZD	13.9 ± 0.8 pA	15	13.1 ± 0.9 pA	15	Cell	LMM	Normal		0.308	0.7523
S5D	WT vs. *Fmr1*$^{-/y}$	3.5 ± 0.6 Hz	15	5.7 ± 0.6 Hz	15	Cell	GLMM	Gamma		2.986	0.0162
	WT: Ctrl vs. ZD	3.5 ± 0.6 Hz	15	4.5 ± 0.8 Hz	15	Cell	GLMM	Gamma		1.413	0.1645
	Fmr1$^{-/y}$: Ctrl vs. ZD	5.7 ± 0.6 Hz	15	5.1 ± 0.6 Hz	15	Cell	GLMM	Gamma		-1.698	0.0991
S5E	WT vs. *Fmr1*$^{-/y}$	0.44 ± 0.04 ms	15	0.40 ± 0.02 ms	15	Cell	LMM	Log-normal		-1.359	0.2128
	WT: Ctrl vs. ZD	0.44 ± 0.04 ms	15	0.54 ± 0.06 ms	15	Cell	LMM	Log-normal		1.773	0.07949
	Fmr1$^{-/y}$: Ctrl vs. ZD	0.40 ± 0.02 ms	15	0.42 ± 0.03 ms	15	Cell	LMM	Log-normal		0.487	0.6192
S5F	WT vs. *Fmr1*$^{-/y}$	2.63 ± 0.15 ms	15	2.50 ± 0.15 ms	15	Cell	LMM	Log-normal		-0.796	0.4488
	WT: Ctrl vs. ZD	2.63 ± 0.15 ms	15	3.14 ± 0.33 ms	15	Cell	LMM	Log-normal		1.61	0.1089
	Fmr1$^{-/y}$: Ctrl vs. ZD	2.50 ± 0.15 ms	15	2.71 ± 0.21 ms	15	Cell	LMM	Log-normal		0.60	0.5413
S6B	WT vs. *Fmr1*$^{-/y}$	-61.7 ± 13.1	65	20.2 ± 5.1	48	Cell	F-test	Linear	Slope	35.05	<0.0001
S6C	WT vs. *Fmr1*$^{-/y}$	25.9 ± 4.5 ms	13	39.5 ± 6.2 ms	16	Cell	Unpaired t-test	Linea		1.78	0.0873
S6D	WT: Ctrl vs. ZD	-61.7 ± 13.1	65	-6.1 ± 12.1	24	Cell	F-test	Linear		6.35	0.0136
	WT: Ctrl vs. ZD	25.9 ± 4.5 ms	13	47.0 ± 7.1 ms	4	Cell	Unpaired t-test	Normal		2.52	0.0480
S6E	*Fmr1*$^{-/y}$: Ctrl vs. ZD	20.2 ± 5.1	48	40.5 ± 11.8	30	Cell	F-test	Linear		3.16	0.0799
	Fmr1$^{-/y}$: Ctrl vs. ZD	39.5 ± 6.2 ms	16	36.4 ± 8.2 ms		Cell	Unpaired t-test	Normal		0.31	0.7634
S7A	WT: Ctrl vs. ZD	0.57 ± 0.16 mV.mV^{-1}	3	0.44 ± 0.13 mV.mV^{-1}	3	Cell	F-test	Linear		0.29	0.7520
S7B	*Fmr1*$^{-/y}$: Ctrl vs. ZD	1.04 ± 0.08 mV.mV^{-1}	4	0.44 ± 0.08 mV.mV^{-1}	4	Cell	F-test	Linear		54.3	<0.0001
S7C	WT: Ctrl vs. AP-5	0.50 ± 0.09 mV.mV^{-1}	3	0.42 ± 0.07 mV.mV^{-1}	3	Cell	F-test	Linear		0.08	0.7738
S7D	*Fmr1*$^{-/y}$: Ctrl vs. AP-5	0.80 ± 0.09 mV.mV^{-1}	5	0.29 ± 0.16 mV.mV^{-1}	5	Cell	F-test	Linear		7.85	0.0060
S8A	WT vs. *Fmr1*$^{-/y}$	173.3 ± 30.7 pA	19	140.6 ± 24.2 pA	18	Cell	Mann-Whitney	Non-normal		137	0.3089
S8B	WT vs. *Fmr1*$^{-/y}$	5.2 ± 0.8 mV	26	4.9 ± 0.8 mV	20	Cell	Mann-Whitney	Non-normal		238	0.6310
S8C	WT vs. *Fmr1*$^{-/y}$	1.6 ± 0.1 ms	26	1.9 ± 0.3 ms	20	Cell	Mann-Whitney	Non-normal		256	0.9342
S8D	WT vs. *Fmr1*$^{-/y}$	56.7 ± 11.3 ms	22	49.1 ± 11.7	20	Cell	Mann-Whitney	Non-normal		192	0.4881
S8E	WT vs. *Fmr1*$^{-/y}$	30.9 ± 5.5 ms	23	35.9 ± 10.1 ms	20	Cell	Mann-Whitney	Non-normal		214	0.7025

Supplementary Table 1: Summary of all statistical tests performed in current study:F-test: Sum-of-least-Squares F-test; Unpaired t-test: Unpaired Student's 2-tailed t-test; Paired t-test: Paired Student's t-test; GLMM: Generalised Linear Mixed-Effects Model; LMM: Linear Mixed-Effects Model; Mann-Whitney: Mann-Whitney U test; Fisher Exact: Fisher's exact contingency test. All data is shown as mean ± SEM and source data are provided as a Source Data file.
