# Supplementary material for: Altered dendritic spine function and integration in a mouse model of fragile X syndrome
Source: Nat Commun. 2019 Oct 23;10:4813. doi: 10.1038/s41467-019-11891-6 (PMC6811549; doi:10.1038/s41467-019-11891-6)
Supplement: Supplementary file 3 — Reporting Summary [file 41467_2019_11891_MOESM3_ESM.pdf]

nature research

Corresponding author(s): *Double-blind peer review submissions: write DBPR and your manuscript number here instead of author names.*

Reporting Summary

Nature Research wishes to improve the reproducibility of the work that we publish. This form provides structure for consistency and transparency in reporting. For further information on Nature Research policies, see Authors & Referees and the Editorial Policy Checklist.

Statistical parameters

When statistical analyses are reported, confirm that the following items are present in the relevant location (e.g. figure legend, table legend, main text, or Methods section).

n/a	Confirmed	
☐	☒	The exact sample size (*n*) for each experimental group/condition, given as a discrete number and unit of measurement
☐	☒	An indication of whether measurements were taken from distinct samples or whether the same sample was measured repeatedly
☐	☒	The statistical test(s) used AND whether they are one- or two-sided<br>*Only common tests should be described solely by name; describe more complex techniques in the Methods section.*
☒	☐	A description of all covariates tested
☐	☒	A description of any assumptions or corrections, such as tests of normality and adjustment for multiple comparisons
☐	☒	A full description of the statistics including central tendency (e.g. means) or other basic estimates (e.g. regression coefficient) AND variation (e.g. standard deviation) or associated estimates of uncertainty (e.g. confidence intervals)
☐	☒	For null hypothesis testing, the test statistic (e.g. *F*, *t*, *r*) with confidence intervals, effect sizes, degrees of freedom and *P* value noted<br>*Give P values as exact values whenever suitable.*
☒	☐	For Bayesian analysis, information on the choice of priors and Markov chain Monte Carlo settings
☐	☒	For hierarchical and complex designs, identification of the appropriate level for tests and full reporting of outcomes
☐	☒	Estimates of effect sizes (e.g. Cohen's *d*, Pearson's *r*), indicating how they were calculated
☐	☒	Clearly defined error bars<br>*State explicitly what error bars represent (e.g. SD, SE, CI)*

Our web collection on statistics for biologists may be useful.

Software and code

Policy information about availability of computer code

Data collection	2 forms of data were collected for this submitted manuscript: primarily electrophysiological trace records and anatomical images. For collection of electrophysiological traces pClamp 10 (Axon Instruments, CA, USA) was used and trace files saved in the .abf file type. For images 3-dimensional image stacks were collected using either STED microscopy (Leica, Germany) as .lif files, or serial block-face scanning electron micrscopy (3View, Gatan, CA, USA) as .tif files.
Data analysis	All data analysis of electrophysiological data was performed with either Stimfit (https://github.com/neurodroid/stimfit) of Clampex (Axon Instruments, CA, USA). All image analysis was performed in the FIJI extension of ImageJ (https://imagej.net/GitHub), where appropriate images were first deconvolved using Huygens Deconvolution software (Scientific Volume Imaging, The Netherlands). Data were collated in Microsoft Excel. All statistical analysis was performed either Linear Mixed-Effects Modelling, which was performed in R (https://www.r-project.org/), or with 2-way ANOVA, Sum-of-least-squares F-test or Student's t-tests, which were performed in Graphpad Prism. All data was tested for normality and the appropriate statistic employed. A full summary of all statistical tests, replicate tested, degree's of freedom, and statistical assumptions is presented in supplementary table 1.

For manuscripts utilizing custom algorithms or software that are central to the research but not yet described in published literature, software must be made available to editors/reviewers upon request. We strongly encourage code deposition in a community repository (e.g. GitHub). See the Nature Research guidelines for submitting code & software for further information.

Data

Policy information about availability of data

All manuscripts must include a data availability statement. This statement should provide the following information, where applicable:

- Accession codes, unique identifiers, or web links for publicly available datasets
- A list of figures that have associated raw data
- A description of any restrictions on data availability

All data will be made available on request

Field-specific reporting

Please select the best fit for your research. If you are not sure, read the appropriate sections before making your selection.

[x] Life sciences [ ] Behavioural & social sciences [ ] Ecological, evolutionary & environmental sciences

For a reference copy of the document with all sections, see nature.com/authors/policies/ReportingSummary-flat.pdf

Life sciences study design

All studies must disclose on these points even when the disclosure is negative.

Sample size	Sample size was determined with power-analysis, following preliminary examination of data. In brief, pilot experiments were performed with minimal biological replicates to determine variability and potential effect size of data. Following this initial assessment further data were collected to satisfy the calculated "n" required based on the observed effect size.
Data exclusions	No data was excluded
Replication	All experiments were performed in successive rounds (see sample size calculation), with a minimum of 2 rounds of experiments performed as routine, up to the number of replicates indicated by power analysis or by previous studies in the lab. The chosen replicate for all individual experiments was assumed as the animal, unless linear mixed effects statistical modeling was employed, which excludes effects of pseudoreplication.
Randomization	All animals were randomly chosen from each litter as they were used prior to gentoyping. It is important to note that there are no outward phenotypes in Fmr1knock-out mice that would potentially bias animal selection towards a particular genotype.
Blinding	All experiments and analysis were performed blind to genotype. As experiments were performed in young postnatal mice (day 11-15, see below) biopsies for genotyping were only collected on the day of experiment. Only once all data collection and analysis for a round of experiments was complete would genotyping be performed and the data unblinded. As such all data was collected in a blind and random fashion.

Reporting for specific materials, systems and methods

Materials & experimental systems

n/a	Involved in the study
x	Unique biological materials
x	Antibodies
x	Eukaryotic cell lines
x	Palaeontology
	x Animals and other organisms
x	Human research participants

Methods

n/a	Involved in the study
x	ChIP-seq
x	Flow cytometry
x	MRI-based neuroimaging

Animals and other organisms

Policy information about studies involving animals; ARRIVE guidelines recommended for reporting animal research

Laboratory animals	All mice used in the current study were of the C57/Bl6J strain (Jackson Labs). Only male mice were used for the current study due to the X-linked nature of the Fmr1 gene. Mice were 11-15 days postnatal for all experiments in this study, as based on the first day pups were observed by handlers defined as postnatal day 0.

Wild animals	N/A
Field-collected samples	N/A
